# A Strategy to Establish a Quality Assurance/Quality Control Plan for the Application of Biosensors for the Detection of *E. coli* in Water

**DOI:** 10.3390/bios7010003

**Published:** 2017-01-03

**Authors:** Nikou Hesari, Nursel Kıratlı Yılmazçoban, Mohamad Elzein, Absar Alum, Morteza Abbaszadegan

**Affiliations:** 1 Department of Civil, Environmental & Sustainable Engineering, Arizona State University, Tempe, AZ 85287, USA; nhesari@gmail.com (N.H.); moelzein@msn.com (M.E.); absar.alum@asu.edu (A.A.); 2 Department of Environmental Engineering, Sakarya University, Serdivan, Sakarya 54187, Turkey; nkiratli@sakarya.edu.tr

**Keywords:** quality assurance/quality control, *E. coli*, MUG substrate, β-d-glucuronides’, drinking water, rapid bacterial detection

## Abstract

Rapid bacterial detection using biosensors is a novel approach for microbiological testing applications. Validation of such methods is an obstacle in the adoption of new bio-sensing technologies for water testing. Therefore, establishing a quality assurance and quality control (QA/QC) plan is essential to demonstrate accuracy and reliability of the biosensor method for the detection of *E. coli* in drinking water samples. In this study, different reagents and assay conditions including temperatures, holding time, *E. coli* strains and concentrations, dissolving agents, salinity and pH effects, quality of substrates of various suppliers of 4-methylumbelliferyl glucuronide (MUG), and environmental water samples were included in the QA/QC plan and used in the assay optimization and documentation. Furthermore, the procedural QA/QC for the monitoring of drinking water samples was established to validate the performance of the biosensor platform for the detection of *E. coli* using a culture-based standard technique. Implementing the developed QA/QC plan, the same level of precision and accuracy was achieved using both the standard and the biosensor methods. The established procedural QA/QC for the biosensor will provide a reliable tool for a near real-time monitoring of *E. coli* in drinking water samples to both industry and regulatory authorities.

## 1. Introduction

Microbiological testing can provide valuable information only if sampling plans and methodology are properly designed and performed. The U.S. EPA states, “compliance monitoring is one of the key components the Agency uses to protect human health and the environment by ensuring that the regulated community obeys environmental laws/regulations through on-site visits by qualified inspectors, and a review of the information EPA or a state/tribe requires to be submitted” [1]. To ensure that compliance monitoring standards are met, several procedures must be followed. Quality Control (QC) of recording, validating, and reporting data are necessary steps to produce complete and scientifically defensible reports. On the other hand, Quality Assurance (QA) programs are also used to evaluate instrument and equipment maintenance and performance as well as the quality of reagents [2].

With emerging sophisticated detection technologies in water quality analysis, the need for incorporating an effective QA/QC plan and the methods of validation increase. Validation is an obstacle in the adoption of new bio-sensing technologies. While such technologies will allow for faster and sensitive detection capabilities, they increase the need for internal quality control and adequately trained personnel to ensure accuracy and proper interpretation of the results. Regulatory approval of molecular methods implies strict QA/QC performance and inter-laboratory validation [3]. Therefore, a validated rapid method to detect indicator bacteria in drinking water is of primary importance for monitoring water quality from the source to the tap.

Appropriate QA/QC measures are necessary for any detection and monitoring system to ensure the reliability of the analytical data generated and increase confidence in the relevance of possible responses. Such data minimizes the probability of false-positive/false-negative responses affiliated with the assay reagents and conditions. Measurements such as complete review of the result’s QA/QC, resampling, and reproducibility of the analysis, and performing more accurate or more precise alternative methods of analysis may be included in the data confirmation process [4].

In our previous study, we identified specific bacterial enzymatic-biochemical signatures that can be used in a custom designed opto-electronic biosensor platform for the detection of *E. coli* and other bacterial cells in water samples [5,6]. The new generations of biosensors rely on bacterial enzymatic responses to specific fluorogenic substrates. The assay is based on using the compound 4-methylumbelliferone glucuronide (MUG), which is hydrolyzed by the specific *E. coli* glucuronidase (GUD) enzyme to yield a fluorogenic product that can be quantified and directly related to the number of *E. coli* cells in water samples. The system is based on measuring the response of bacterial enzymatic machinery to the added specific fluorogenic substrates.

Over the last decade, new methods for rapid detection of waterborne bacterial pathogens have emerged; however, these methods require considerable processing including sample concentration. Our innovative method requires minimum processing and only detects viable bacterial cells. It relies on a unique reaction chemistry that enhances the quality and the intensity of measureable signals providing a near real-time assay. Rapid assays to estimate the GUD activity of *E. coli* have been performed without any cultivation steps where direct measurements of GUD activity were successfully applied to river, sea, and waste water samples [7,8,9,10]. However, current procedures are laboratory-based and require bench-top fluorometers for the measurement of fluorescence resulting from the enzyme–substrate reaction. The biosensor used in this study only detects viable and viable but non-culturable (VBNC) *E. coli* in the water sample between two to four hours and can be customized in handheld or a bench-top fluorometer format [5].

The present study attempts to establish the procedural QA/QC for the new biosensor platform, and the ability to demonstrate acceptable precision and accuracy of *E. coli* detection in drinking water samples. A guideline was adopted to examine an array of potential changes in chemical, physical, and biological conditions impacting the accuracy of bacterial detection outcome using the biosensor methodology. The strategy used in this study included the importance of different temperature, sample holding time, *E. coli* strains and concentration, dissolving agents, water versus Phosphate Buffered Saline (PBS), salinity and pH effects on the assay, quality of MUG substrates from different suppliers, and different environmental water samples. These criteria were evaluated for the assay optimization to establish the QA/QC requirements for the detection of *E. coli* in drinking water samples. It is important to note that only under an established procedural QA/QC, the biosensor will provide a reliable tool for a near real-time monitoring of *E. coli* in drinking water samples.

In our previous study, some significant factors such as the sensitivity (detection limit) and specificity of the assays were tested for both tap and environmental waters and have shown a sensitivity threshold of seven *E. coli* cells per reaction vial. This demonstrated the highest sensitivity of the BDS1000 biosensor that was better or comparable to the sensitivity of other hand-held fluorescence detector [11]. This study only focuses on the parameters effecting the reaction cocktail and QA/QC procedure of the assay. The effect of water contaminant interferences such as heavy metals on assay sensitivity with lower concentrations of bacteria have been documented in our previous publications [5,6] which focused on the application of this innovative method in environmental water, including water from lakes that receive regular application of copper sulfate for algal control. The result showed that no significant interference from non-GUD sources in the water samples existed.

## 2. Materials and Methods

A study was conducted to evaluate the effects of experimental variables on the quality of the data and to lay out an appropriate QA/QC plan. All experiments were conducted under laboratory conditions using aseptic techniques. To rapidly detect viable *E. coli* in water samples, their GUD response to the added specific fluorogenic substrates (MUG) was measured using the resulting 4-MU fluorescence signals upon its hydrolysis by the enzyme-substrate reaction. The fluorescence signals were directly measured without any cultivation steps by the customized BDS1000 fluorescence detector.

### 2.1. Stock Culture Preparation

Pure cultures of *E. coli* obtained from American Type Culture Collection (ATCC) were grown in Tryptic Soy Broth (TSB) (Becton Dickinson, Sparks, MD, USA). Log phase bacterial stocks were prepared by incubating the bacterial suspension at 37 °C in a C24 shaker-incubator (New Brunswick Scientific, Edison, NJ, USA) at 150 rpm. The log-phase bacterial cultures were stored at 4 °C for at least 24 h before their use for the assays. Bacterial stocks were diluted in 0.1X PBS in a range of 10–10^8^ CFU/mL.

### 2.2. Confirmation of E. coli Strains

Collilert-18 (IDEXX, Westbrook, ME, USA) has been applied as a GUD validation tool prior to the assay in this research. Collilert-18 is a new standard in coliform/*E. coli* detection, which is known as QC procedure based on IDEXX’s patented Defined Substrate Technology (DST). When *E. coli* metabolizes Collilert-18’s nutrient-indicator, MUG, the sample also fluoresces. It is reported that the method is able to detect a single viable coliform or *E. coli* per sample and eliminates false positive detection of non-target organisms [5,6,7,8,9,10,11,12]. For each test, contents of one pack of Collilert-18 were added to a 100 mL sample in a sterile, transparent, non-fluorescing vessel which was then capped and shaken. One mL of the overnight culture of *E. coli* stock was added to the 100 mL of the sample and then incubated at 37 °C for 18 h to confirm GUD activities of the selected isolates.

### 2.3. Culture-Based Assays

At the start and completion of each assay, *E. coli* concentration was measured by the Membrane Filtration (MF) technique using a 0.45 µm membrane (Millipore SAS, Billerica, MA, USA) and Brilliance (Oxoid Ltd., Basingstoke, UK) or mEndo (Becton, Dickinson and company) media followed by incubation at 37 °C for 24 h. This step was performed to obtain CFUs before and after the assay and to ensure bacterial viability and culturability.

### 2.4. Different Assay Conditions: Temperature, MUG, and E. coli

*E. coli* cultures kept at 4 °C for the one to seven days prior to use for the assay. Also, samples were stored both at room temperature (~24 °C) and at 37 °C. For every assay, samples were incubated at 37 °C in a hot plate in 10 min intervals prior to each measurement.

Assays were performed in triplicate by simultaneously processing three aliquots of *E. coli* suspension in three separate reaction vials and examined using the biosensor with the substrate purchased from different MUG suppliers, Sigma Chemical Co. (St. Louis, MO, USA), EMD Millipore (Billerica, MA, USA) and Bioworld (Dublin, OH, USA) in order to study the quality and functionality of the substrates. For the comparison, the substrate was dissolved in Dimethyl Sulfoxide (DMSO), purchased from Mallinckrodt Baker Inc. (Paris, KY, USA) and in ethanol according to the MUG suppliers’ preparation instruction. In addition, *E. coli* was diluted in 10 mL of 0.1, 0.5, and 1X PBS at pH 7.3 and the results were compared to GUD activities. Furthermore, two additional *E. coli* strains, ATCC 35218 and 1177 were compared with the reference strain ATCC 25922.

### 2.5. Effect of pH and Salinity

Alkalinity of the sample was increased by adding NaOH to N-[2-hydroxyethyl]piperazine-N′-[2-ethanesulfonic acid] Buffer (HEPES) and adjusted to pH 8 or 9 before testing. Furthermore, samples were prepared by dissolving 5 g/L (0.5%) NaCl in the water sample, and the results were compared with the samples without adding NaCl. Each set of assays consisted of 3.7 mL of a representative sample containing 0.5% NaCl.

### 2.6. Blanks and Reference Instrument

For each subsequent sample concentrate, including blank or spiked samples, fluorescence intensity measurements were performed. The fluorescence intensity values were averaged and compared to the blank. The results have been compared and evaluated with the performance of the reference instrument Aqualog® bench-top fluorometer (Horiba, Kyoto, Japan), the only simultaneous absorbance and fluorescence system designed for water quality analysis that measures both absorbance spectra and fluorescence excitation-emission matrices.

### 2.7. Biosensor

A biosensor was assembled inhouse by obtaining optical and spectrometer components (Model # HR 2000, Ocean Optics, Dunedin, FL, USA). The xenon light source was used to provide filtered excitation light at specific wavelengths to allow single excitation, single emission detection of a specific fluorophore. The specificity of the detection was ensured by excitation light spectrum with a ±10 nm range around the peak. This allowed all fluorescence assays to be carried out at a single excitation wavelength (350 ± 10 nm).

## 3. Results and Discussion

**Figure 1:**
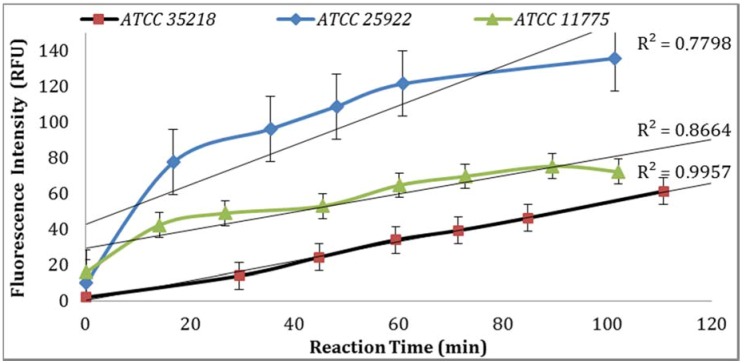
Time series of hydrolysis of MUG by different strains of *E. coli* (100 CFU/mL).

Three *E. coli* strains were evaluated for GUD activities, and the strain (ATCC 25922) with the highest GUD activity was selected for further studies (Figure 1). To develop QA/QC framework, the performance of the biosensor platform was studied and validated under varying experimental conditions. The key components evaluated to establish a set of QA/QC for the detection of *E. coli* in water samples included: temperature, sample holding time, *E. coli* strains and concentration, substrates, dissolving agents, water versus buffer solution, salinity and pH effects on the assay, quality of substrates from different suppliers of MUG, and different environmental water samples. Other considerations included: substrate and reagents new lot examination, sample blanks (method blank and positive and negative controls), validation methods, reference instruments, equipment quality control (annual calibrations), and lab records and documentation. The optimization steps were performed to attain high specificity and sensitivity as measured by GUD enzymatic activity under the established procedural QA/QC plan. To ensure the accuracy and reliability of the data, sterility controls were included for every batch of culture media and buffers used. In addition, growth controls were included for every batch of culture media used to ensure media quality.

Enzymatic activities are subject to the physiological state of bacteria under their nutritional status and stress conditions, a fraction of cells may gradually lose its culturability, although remaining metabolically active. Garcia-Armisen et al. [7] and Togo et al. [13] hypothesized that VBNC cells under stressful conditions—such as nutritional stress and increased sunlight effects, low turbidities, pressure, high or low salinity—have higher GUD activities [14,15,16].

### 3.1. Confirmation of E. coli Strains

GUD activities in three different strains of *E. coli*, ATCC 25922, 35218, and 11775 were validated using the Collilert-18 kit. When *E. coli* metabolized Collilert-18’s nutrient-indicator, *ortho*-Nitrophenyl β-galactoside (ONPG), the sample turns yellow and fluoresces under UV light. The findings were in agreement with the previous research [16,17] and proved that *E. coli* 25922 produces the highest GUD activities (Figure 1).

### 3.2. Assay Conditions

#### 3.2.1. Effect of Temperature

**Figure 2:**
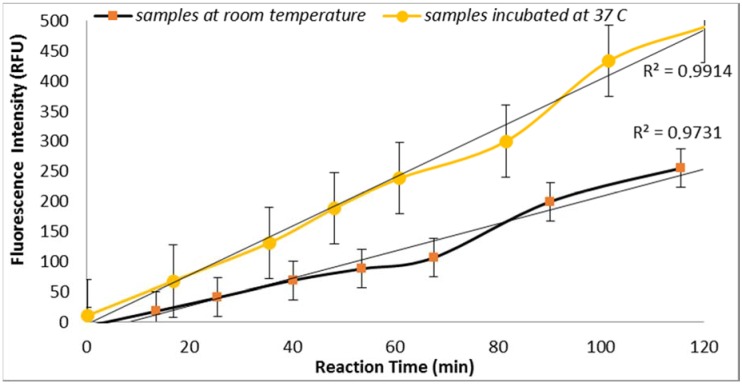
Effect of temperature of incubation on GUD activities in *E. coli* (100 CFU/mL) as measured by fluorescence intensity.

*E. coli* GUD activities are sensitive to the temperature of incubation as supported by the results obtained from the assays performed using two sample aliquots containing 100 CFU/mL of *E. coli* incubated at room temperature and 37 °C (Figure 2). This finding is in agreement with the previous study reported by Caruso et al. that “the specificity and selectivity of the enzyme assays towards *E. coli* are strongly related to the temperature of incubation” [18]. This finding is also in agreement with the previous studies that have shown increased selectivity associated with the higher temperature which may have inhibited the growth of injured or stressed cells [18].

#### 3.2.2. MUG Quality

**Figure 3:**
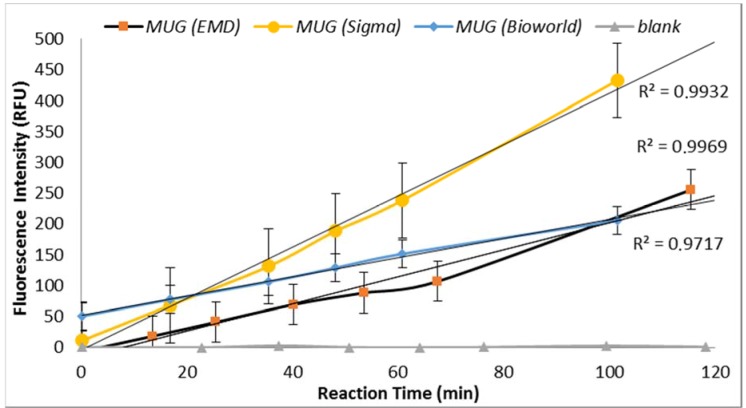
Comparison of GUD response to MUG obtained from different suppliers. Note: no increasing trend in the relative fluorescence units (RFU) was noted in the control samples. The data points are the average of three replicates.

Under similar assay conditions, enzyme-substrate reaction with MUG obtained from three suppliers (Sigma Chemical Co., St. Louis, MO, USA; EMD Millipore, Billerica, MA, USA and Bioworld, Dublin, OH, USA) yielded significantly different fluorescence intensities (Figure 3). MUG purchased from Sigma in May 2014 (lot# BCBH7903V) resulted in non-reproducible data. MUG purchased from Bioworld produced similar fluorescent intensity as previously obtained by MUG purchased from Sigma before May 2014 (Figure 3). Therefore, the QA/QC plan for an enzyme-based biosensor should include enzyme-substrate reactivity level determination. Furthermore, a blank sample (all reagents with no *E. coli*) was included for each set of samples and no increasing trend in the relative progression of GUD activities was noted in all blank samples.

#### 3.2.3. Effect of Dissolving Agents (Solvents for Enzyme Substrate) and Buffer Strength

**Figure 4:**
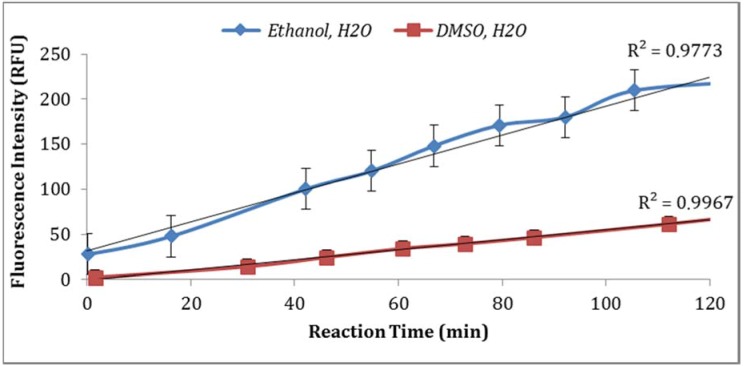
Impact of different solvents for dissolving MUG on assay sensitivity (fluorescence intensity).

**Figure 5:**
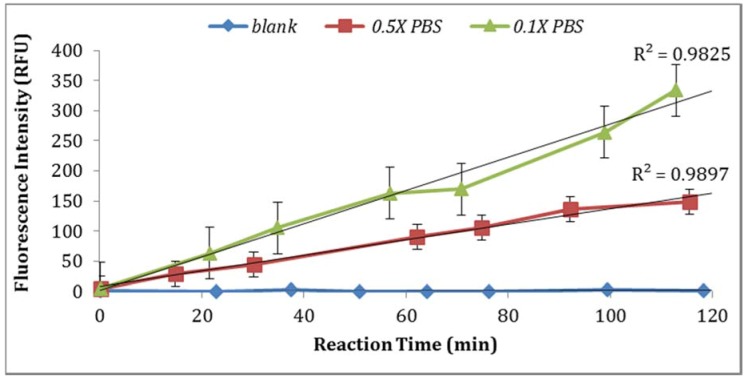
Impact of buffer strength on fluorescence intensity.

**Figure 6:**
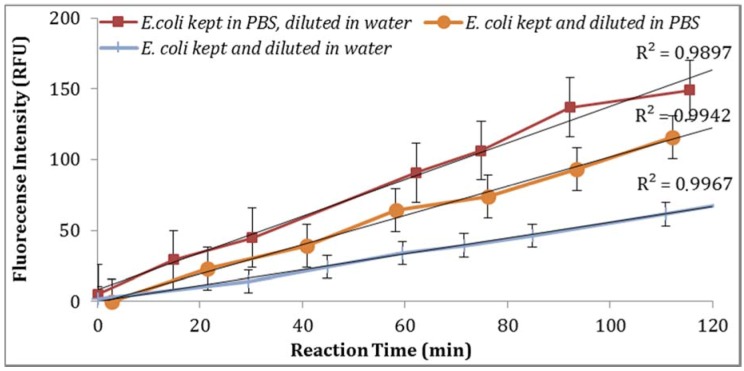
Effect of storage condition of *E. coli* on GUD activities.

Two solvents (ethanol and DMSO) were compared for their impact on the assay sensitivity using the sample aliquots from the same *E. coli* stock. Using DMSO to dissolve MUG resulted in lower fluorescent intensity than ethanol (Figure 4). In addition, the impact of PBS strength on fluorescence intensity was investigated. The assay using 0.1X PBS yielded higher enzymatic activity compared to the assay using 0.5X PBS (Figure 5). Moreover, the impact of *E. coli* stored in PBS or tap water at 4 °C with subsequent dilutions in PBS or tap water under the same laboratory conditions was examined. As illustrated in Figure 6, storing *E. coli* in PBS with dilutions in water resulted in higher fluorescence measurements (Note: Samples diluted in 0.1X PBS and spiked tap water samples contained 0.5% NaCl).

#### 3.2.4. Holding Time

Prior to each assay, *E. coli* cultures were stored overnight at 4 °C to allow cells to reach their stationary/starvation phase reflecting the physiological state of environmental isolates. The high levels of GUD activity observed in the cultures may indicate that their starved metabolic state leads to an increase in bacterial enzymatic activities by hydrolyzing the fluorogenic substrate rapidly. Caruso et al. [18] reported that the full development of enzymatic activities start at the lag phase and is required for the enzyme expression.

Enzyme-substrate and HEPES buffer storage time play a significant role in the enzymatic assay and generation of fluorescence signals. The quality of MUG working stock decreased after one week of storage, where crystallization of MUG in the working stock was observed. Therefore, it is suggested that working stocks of MUG should not be used after 3–5 days of storage. MUG and HEPES are light-sensitive chemicals and should be considered in their storage and use conditions. In summary, all QC requirements must be met for each new lot of reagents and standards prior to use in the assay to ensure the reliability of the results (Table 1).

#### 3.2.5. Effect of pH and Salinity on the Intensity of Fluorescence Signal

It is observed that adding salt impacts the microbial growth; therefore, this factor has been evaluated in this study. Moreover, the role of alkalinity in the GUD assays for marine waters has been reported previously. However, the present study investigates this parameter in drinking water [18].

**Figure 7:**
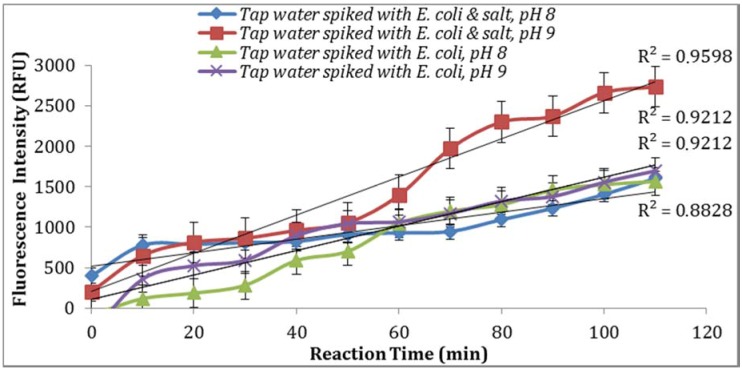
Effect of pH and salinity on the fluorescence signal in the biosensor assay. Note: Spiked water samples contained 1000 CFU/mL *E. coli*.

As seen in Figure 7, parallel assays using the sample aliquots from the same *E. coli* stock yielded higher enzymatic activity at pH 9. This is in agreement with previous studies reported by Caruso et al. [18] where the addition of NaOH before the spectro-fluorometric measurement entails an increase in fluorescence. Furthermore, a pH of 9 or 10 were suggested by Geary et al. [19] and Hoppe et al. [20] as the optimum pH value at which MU reaches its peak of fluorescence intensity. In addition, supplementing the reaction with 0.5% NaCl resulted in an increase in the intensity of fluorescence signal (Figure 7).

Drinking water distribution systems are required to maintain disinfectant residuals to ensure the microbial quality of water delivered to municipal customers. Disinfectants are known to interfere with GUD and MUG [21]; however, residual disinfectants in water samples are neutralized prior to microbial analysis. From the perspective of application of this biosensor technology in drinking water, the residual disinfectant is irrelevant. Therefore, the impact of disinfectants on the performance of this technology was not tested.

**Figure 8:**
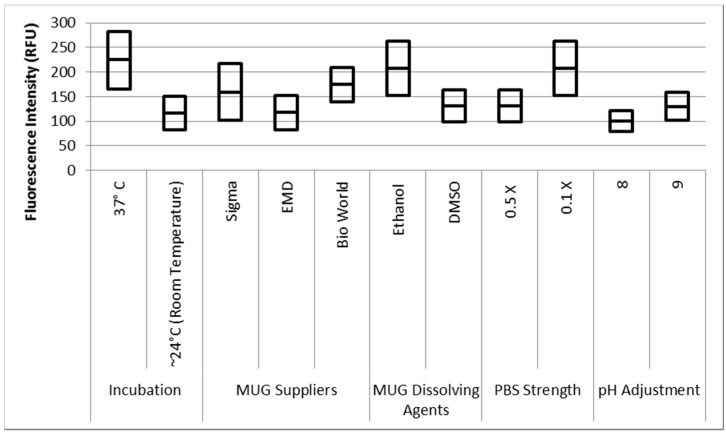
Effect of different assay conditions on fluorescence intensity. Note: The results have been obtained based on three replicates of each sample from three independent experiments; the units are RFU (arbitrary units) samples contained 100 CFU/mL *E. coli*.

The data was statically analyzed to compare the effect of different reagents concentrations and assay conditions on GUD activities. For each variable, the average GUD activity [with lower and upper 95% Confidence Intervals (CI)] is presented as box plot in Figure 8. As far as the impact of temperature of incubation, GUD activity increased remarkably over time in samples incubated at a higher temperature (Figure 8). For example, the average fluorescence intensity for samples incubated at 24 °C and 37 °C were 116 and 225 relative fluorescence units (RFU), respectively. A similar impact was recorded for the dissolving agents, with 131 and 208 average RFU for DMSO and ethanol, respectively. Other variables that affected fluorescence intensity were pH of the reaction and PBS strength used in the assay. The pHs 8 and 9 resulted in 100 and 130 RFU, respectively, and RFU of 131 and 208 were recorded for PBS strength at 0.5X and 0.1X respectively. Additionally, MUG from different sources (EMD Biosciences, San Diego, CA, USA; Bioworld, Dublin, OH, USA and Sigma-Aldrich; St. Louis, MO, USA) showed variable levels of fluorescent activity when tested at the same concentrations. The obtained data for all the assay conditions provided the desired linearity (R^2^ = 0.90 or higher); otherwise, the assays were repeated. For establishing the procedural QA/QC for every assay, the baseline conditions used were MUG from Bioworld dissolved in ethanol and samples incubated at 37 °C during the assay. The samples were diluted in 0.1X PBS and pH of the reaction was adjusted to 9 using HEPES buffer.

## 4. Conclusions

A set of QA/QC requirements has been established for the detection of *E. coli* in drinking water samples using enzyme-based biosensor BDS1000. The enzyme-substrate reaction was proven to be specific for the detection of *E. coli* under the laboratory experimental conditions performed. The proposed technique provides significant time saving over the existing methods with comparable cost. The biosensor method was validated using standard biochemical and microbiological procedures, and was proven to provide accurate and reliable data with acceptable precision under the established plan. The proposed procedural QA/QC plan will ensure the quality and reliability of the data that can be used by both industry and regulatory authorities for near real-time monitoring of *E. coli* in drinking water samples.

The BDS1000 biosensor device can be improved and customized for either laboratory or field applications. The main limitation of the device is its low sample volume capabilities and throughput that can be analyzed at a time; however, this issue can be addressed by investigating sample processing and sample concentration to allow a minimum of 100 mL to be tested for the presence of indicator bacteria in drinking water.

Based on the lessons learned from this study, it is only under an established procedural QA/QC that the biosensor can be used as a reliable tool for near real-time monitoring of *E. coli* in drinking water samples. The lack of a proper procedural QA/QC plan may contribute to a false negative result that may lead to wrong decision making and adverse effects on public health.

## Figures and Tables

**Table 1:** Parameters for QA/QC of the biological and chemical factors for the biosensor assay.

Reagents and Standards		
Bacterial Cultures	Reagents-substrates/enzyme	Buffers–HEPES
QC for each media reference strain–ATCC	QC for each batch enzyme-substrate	QC for each batch pH verification buffering capacity
